# Antioxidant Supplementation in Renal Replacement Therapy Patients: Is There Evidence?

**DOI:** 10.1155/2019/9109473

**Published:** 2019-01-15

**Authors:** Vassilios Liakopoulos, Stefanos Roumeliotis, Andreas Bozikas, Theodoros Eleftheriadis, Evangelia Dounousi

**Affiliations:** ^1^Division of Nephrology and Hypertension, 1st Department of Internal Medicine, AHEPA Hospital, School of Medicine, Aristotle University of Thessaloniki, Thessaloniki, Greece; ^2^Department of Nephrology, Medical School, University of Ioannina, Ioannina, Greece

## Abstract

The disruption of balance between production of reactive oxygen species and antioxidant systems in favor of the oxidants is termed oxidative stress (OS). To counteract the damaging effects of prooxidant free radicals, all aerobic organisms have antioxidant defense mechanisms that are aimed at neutralizing the circulating oxidants and repair the resulting injuries. Antioxidants are either endogenous (the natural defense mechanisms produced by the human body) or exogenous, found in supplements and foods. OS is present at the early stages of chronic kidney disease, augments progressively with renal function deterioration, and is further exacerbated by renal replacement therapy. End-stage renal disease patients, on hemodialysis (HD) or peritoneal dialysis (PD), suffer from accelerated OS, which has been associated with increased risk for mortality and cardiovascular disease. During HD sessions, the bioincompatibility of dialyzers and dialysate trigger activation of white blood cells and formation of free radicals, while a significant loss of antioxidants is also present. In PD, the bioincompatibility of solutions, including high osmolality, elevated lactate levels, low pH, and accumulation of advanced glycation end-products trigger formation of prooxidants, while there is significant loss of vitamins in the ultrafiltrate. A number of exogenous antioxidants have been suggested to ameliorate OS in dialysis patients. Vitamins B, C, D, and E, coenzyme Q10, L-carnitine, a-lipoic acid, curcumin, green tea, flavonoids, polyphenols, omega-3 polyunsaturated fatty acids, statins, trace elements, and N-acetylcysteine have been studied as exogenous antioxidant supplements in both PD and HD patients.

## 1. Introduction

Every substance that can accept electrons is defined as oxidant, while a compound donating electrons is considered a reductant. Subsequently, a chemical process that includes loss of electrons is defined as oxidation, while reduction is a chemical reaction in which a compound gains electrons. When an oxidant gains electrons, it triggers the oxidation of another compound, and when a reducing agent donates its electrons, it causes the reduction of another substance. Therefore, an oxidation reaction is always accompanied by a reduction process and vice versa. These chemical reactions are called redox reactions. “Oxidant” and “reductant” are biochemical terms, and in cells and tissues, they should be replaced with the terms “prooxidant” and “antioxidant,” respectively. The redox potential or redox state is defined as the ratio between prooxidant and antioxidant agents, and under pathologic conditions, this state can be altered towards redosis or oxidosis. Oxidation results in production of reactive oxygen species (ROS). Although ROS are associated with several harmful events, they are also essential for cellular growth and proliferation. [1]. The disruption of balance between production of ROS and antioxidant systems is termed oxidative stress (OS) [2]. Based on this OS paradox, abundant antioxidants in a cellular environment might scavenge an excessive amount of ROS and therefore inhibit their beneficial role in stimulating important biochemical reactions, crucial for cell homeostasis [3]. The two-sided disruption of balance of the redox state to either excessive reduction or oxidation results in injury and damage of the biological systems [4].

Antioxidants are either endogenous (the natural defense mechanisms produced by the human body) or exogenous, found in food and supplements. Endogenous antioxidants can be either enzymatic or nonenzymatic, including water- or fat-soluble molecules.  Table 1 summarizes the numerous biomarkers of OS, including both oxidants and antioxidant molecules. Since excessive loading of antioxidants might have damaging results in biological systems, antioxidant therapy for patients at high risk for OS should be carefully designed.

Chronic kidney disease (CKD) is a worldwide health problem which has taken the form of an epidemic during the past decades. Its increasing incidence rates and the heavy associated cardiovascular (CV) burden [5] cannot be solely explained by traditional risk factors. OS is highly prevalent in uremia and is considered as an important pathogenetic mechanism and a novel nontraditional risk factor for all-cause and CV mortality in these patients [2, 6]. OS is present even at the early stages of CKD [7], augments in parallel with the progression of the disease to end-stage renal disease (ESRD) [8], and is further exacerbated in hemodialysis (HD) patients [9, 10]. ESRD patients on peritoneal dialysis (PD) manifest significantly enhanced OS compared to nondialysis uremic patients, but lower than HD patients [11].

A number of interventions have been suggested to ameliorate OS in dialysis patients [12]. This review is aimed at presenting the available data regarding the exogenous administration of antioxidants and their possible protective effects on renal replacement therapy (RRT) patients.

## 2. Renal Replacement Therapy and OS

Several factors are implicated in the pathogenesis of OS in HD and PD. ESRD patients commonly have multiple comorbidities related to excessive production of prooxidants, like hypertension, dyslipidemia, diabetes mellitus (DM), vascular calcification, and old age [13]. Chronic inflammation and malnutrition that usually accompany dialysis patients are well-known triggers of ROS production [14]. RRT-related factors also contribute to formation of prooxidants. Due to blood exposure to bioincompatible dialysate, dialyzers, use of heparin, and intravenous iron administration, white blood cells and platelets are activated, leading to acute production of ROS, within minutes of HD session initiation. Each HD session causes a 14-fold increase in circulating ROS levels [15–17]. In PD, the bioincompatibility of PD solutions, including high osmolality, elevated lactate levels, low pH, and accumulation of advanced glycation end-products (AGEs) trigger formation of prooxidants, while there is significant loss of vitamins in the ultrafiltrate [11].

Besides formation of prooxidant molecules, patients on RRT are characterized by significant depletion of antioxidant defense mechanisms, due to a number of reasons: both the traditional, strict dietary restrictions and the malnutrition status that usually accompanies ESRD patients are characterized by limited consumption of fruits and vegetables and thus poor intake of antioxidants such as vitamins C, D, and E [18], while treatment with both PD or HD has been associated with loss of vitamins and trace elements [19–21]. Therefore, it has been hypothesized that administration of several exogenous antioxidants might protect RRT patients from OS-derived cardiovascular morbidity, inflammation, and mortality.

## 3. Exogenous Antioxidant Supplementation in HD and PD Patients

### 3.1. Vitamin E (Alpha-Tocopherol)

#### 3.1.1. Biological Structure and Role

Vitamin E is formed by eight lipid-soluble compounds, including four tocotrienols and four tocopherols. Of these eight isomers, alpha-tocopherol (a-tocopherol) is the only form of vitamin E that is abundantly found in food, maintained in the human body, and is the most biologically active compound. Vitamin E has been identified as the only biological specific free ROS scavenger with the capacity to abrogate peroxidation of cells and molecules by terminating chain reactions. Moreover, through its antioxidant properties, vitamin E acts beneficially on human health, with alleged implications including decrease in various chronic, degenerative diseases' progression, like Alzheimer's disease, cancer, atherosclerosis, CV disease (CVD), allergies, and nonalcoholic fatty liver disease. Although recommendations for dietary intake of vitamin E have long been established, the majority of the population worldwide has suboptimal vitamin E status. In the last decade, there is a growing body of evidence suggesting not only traditional but also novel, unexpected molecular antioxidant properties of vitamin E, and it is hypothesized that the recommended dietary allowance of this vitamin might be reassessed in the following years [22]. During catabolism of vitamin E in the liver, side-chain oxidation leads to formation of bioactive long-chain metabolites. In the last few years, several investigators have increasingly highlighted the crucial role of these vitamin E long-chain metabolites as biological active compounds exerting effects on inflammation, cell apoptosis, lipid metabolism, and proliferation, different from that of their precursors [23, 24]. Moreover, a recent study showed that compared to healthy controls, HD patients presented a defective metabolism of vitamin E, including decreased production rate and subsequent reduced plasma levels of long-chain metabolites and compounds [23]. Galli et al. measured plasma levels of vitamin E metabolites (carboxyethyl hydroxychromans (CEHC)) in patients with CKD stages 3 and 4, HD subjects, and healthy controls and found that the progressive deterioration of renal function was accompanied by a parallel, exponential rise in plasma CEHC concentration [25]. Similarly, two other studies showed that compared to healthy controls, chronic HD patients were characterized by dysregulated vitamin E metabolism and enhanced accumulation of vitamin E metabolites (CEHC) in plasma and serum [26, 27]. The authors of these studies suggested that a higher daily vitamin E intake might be recommended to HD patients.

Lipid peroxides such as malondialdehyde (MDA) cause severe deformation of the erythrocytic membrane, cell apoptosis, and hemolysis. A-tocopherol bonds tightly in lipoproteins and is the most powerful chain-breaking antioxidant in biological membranes of white and red blood cells. The pathophysiological mechanisms underlying the antioxidant effect of vitamin E include preserving the stability and homeostasis of cell membranes, preventing RBC membrane phospholipids from oxidative damage caused by circulating ROS and lipoperoxides, and activation of cell response and defense against OS [28]. Moreover, a-tocopherol may exert an antiatherogenic effect, since it ameliorates the oxidation of low-density cholesterol (LDL), the first and crucial step towards atheromatosis [29, 30].

#### 3.1.2. In Vitro Studies and Animal Models

Bioincompatibility of HD dialyzers, dialysate, and intravenous (i.v.) medications (e.g., iron) trigger activation of polymorphonuclear neutrophils (PMNs) and subsequently ROS production. Several in vitro studies reported the beneficial antioxidant effect of vitamin E supplementation on HD patients' white blood cells (WBCs). Lubrano et al. showed that oxidation response of WBCs was suppressed and their membranes were more stable after vitamin E administration in 10 HD patients, [31], while MDA levels of WBCs were significantly decreased to normal values [32]. Likewise, Hodkova et al. examined the activation of PMNs from the blood of 7 maintenance HD patients, after sessions with i.v. iron and oral vitamin E, and showed that a daily oral intake of 200 mg vitamin E for a week can successfully ameliorate the iron-mediated activation of PMNs, a well-known precursor of OS [33]. Although vitamin E is an antioxidant agent, it has been reported as cytotoxic to the human mesothelium, causing irreversible cell damage [34]. To examine the effect of vitamin E on lipid oxidation and stability of the peritoneal membrane, PD rats were dialyzed with peritoneal solutions enriched with vitamin E. Although vitamin E supplementation caused a significant reduction in peritoneal MDA levels, the structure of the peritoneum was significantly altered and the permeability to protein and glucose significantly increased [34]. Therefore, despite its antioxidant protective role, vitamin E may cause severe structural and functional injuries in the human peritoneum.

#### 3.1.3. Vitamin E for Renal Anemia Improvement

Although vitamin E is a well-known and powerful antioxidant agent, its effect on ESRD-associated anemia remains controversial. In dialysis patients, MDA levels of erythrocytes reflect oxidation of their membranes and are directly associated with anemia status [35, 36]. Since vitamin E can reduce red blood cells' (RBC) MDA levels, it was reasonable to hypothesize that a-tocopherol intake could improve renal anemia. Giardini et al. treated 19 stable HD patients with intramuscular administration of 300 mg/day a-tocopherol for 15 days, measured the fatty acid composition and vitamin E and MDA levels of RBCs before and after the supplementation, and found that administration of a-tocopherol resulted in reduced lipoperoxidation of erythrocytes' membranes and improvement of anemia [37]. Another study with a similar design (intramuscular daily administration of a-tocopherol 300 mg/day for 15 days in 9 HD patients) showed that after treatment, RBC levels of vitamin E increased significantly; lipid peroxidation of erythrocytes, assessed by RBC MDA levels, were significantly decreased and anemia significantly improved [38]. Similarly, Ono reported that HD patients that were supplemented with an oral, daily high dosage of 600 mg a-tocopherol for one month showed a significant increase in serum and RBC vitamin E levels, increase in hemoglobin levels, and decrease in RBC osmolarities, compared to untreated patients. The authors of the study suggested that the beneficial effect of vitamin E supplementation in correcting uremic anemia was attributed to the reduction in RBCs' osmotic fragility resulting in stable cell membranes [39]. In another study, 12 HD patients treated with erythropoietin (EPO) for anemia management were divided to either additional everyday oral intake of 500 mg/day of vitamin E or no intake for 6 months. After the study period, supplementation of vitamin E resulted in a significant decrease in lipid peroxidation status (assessed by serum MDA levels), improvement of renal anemia status, and decreased EPO requirements [40]. The effect of combined therapy with EPO and vitamin E in children on HD was examined in another study. Ten of them were initially treated with EPO for 2 weeks and then with a combination of EPO and vitamin E (15 mg/kg/day per OS) for another 2 weeks. Compared to monotherapy with EPO, the combined treatment with vitamin E resulted in a significant decrease in the ratio of oxidized to reduced glutathione (GSSG/GSH) and a significant improvement of anemia status [41]. Although iron supplementation is necessary for renal anemia management in dialysis patients, it is well-established that intravenous iron administration during HD triggers formation of lipid peroxides and OS. Roob et al. showed that a single oral dose of 1200 IU of vitamin E before the HD session can successfully ameliorate the iron-mediated OS [42]. Another study showed that daily oral supplementation with 400 IU of a-tocopherol for 2 months in a cohort of 11 HD patients doubled serum a-tocopherol levels and increased postdialysis hemoglobin levels but had no effect on OS status [43]. Therefore, it could be hypothesized that vitamin E could exert a beneficial effect on renal anemia, independently of its antioxidant role.

In disagreement with these findings, several investigators found no effect of vitamin E on anemia status of HD patients. A randomized, prospective, double-blind study was conducted by Sinsakul et al., to investigate the possible beneficial effect of vitamin E on anemia status of chronic HD patients. Thirty-five stable, chronic HD patients were randomized to either 400 IU of oral vitamin E twice daily for a period of 20 weeks or placebo. Although serum vitamin E levels were significantly increased in the treated group compared to the placebo, vitamin E supplementation had no effect on the anemia status of HD patients [44]. Similarly, Lillo-Ferez et al. found that oral administration of a-tocopherol at a high dose (600 mg/day) for 30 days had no effect on anemia status in a cohort of 10 chronic HD patients [45]. Another study with a similar design showed that, in a group of 24 chronic HD patients, hemoglobin and EPO levels remained unchanged after 3 months of daily treatment with 400 mg oral a-tocopherol [46].

#### 3.1.4. Vitamin E as Antiatherogenic Agent

Large population-based studies supported that a high dietary intake of vitamin E was associated with a significantly lower risk of coronary heart disease in both men and women [29, 30]. However, a number of interventional studies failed to show any beneficial effect of vitamin E intake on progression of atherosclerosis [47]. A randomized, placebo-controlled, multicenter study included 27 maintenance HD patients from 4 dialysis units that were randomized to oral intake of 800 IU of a-tocopherol daily or placebo for 2 years. Oxidation of proteins, lipids, and AGEs was measured at the end of the study period. Oral administration of a-tocopherol failed to show any protective antioxidant effect [48]. In agreement with these results, Islam et al. treated 17 PD, 16 HD patients, and 16 healthy age/gender-matched controls with oral a-tocopherol (800 IU/day) for 12 weeks. At the end of the study period, although the lipid profile of all three groups remained unchanged, susceptibility of LDL cholesterol to oxidation was significantly decreased in both dialysis groups. Vitamin E supplementation increased LDL a-tocopherol concentrations (controls = 94%, HD = 94%, and PD = 135%), indicating that the possible antioxidant effect of vitamin E was significantly greater in patients on PD subjects than on HD or healthy controls [49]. Since oxidation of LDL is the first step in the pathogenesis of atherosclerosis, a-tocopherol supplementation, according to these results, might exert both antioxidant and antiatherogenic effects at least on PD patients. Moreover, Giray et al. showed that 600 mg/day per OS treatment with vitamin E for 14 weeks in HD patients decreased the risk of OS-induced CVD [50].

There is accumulating evidence that vitamin E attenuates OS in RRT patients. Inal et al. examined the effect of vitamin E on endogenous antioxidant systems and lipid peroxidation status in 46 maintenance HD patients, divided in 3 groups: 10 untreated patients, 36 receiving EPO (100 U/kg) thrice weekly for 3 months, and 36 treated with a combination of EPO at a 50% lower dosage and 300 mg/day of oral vitamin E for 3 months. Compared to the two other groups, those treated with the combination of EPO and vitamin E had significantly decreased serum MDA levels and significantly improved antioxidant status—assessed by superoxide dismutase (SOD) and catalase (CAT) activities [51]. Galli et al. treated 7 stable HD patients with oral intake of 800 mg/day of a-tocopherol for 3 weeks and showed that compared to baseline, after the treatment period, the lipid peroxidation status was significantly decreased [52]. In another study, a low dose of oral 300 mg vitamin E thrice weekly also inhibited OS-derived DNA injury in dialysis patients on HD or PD [53]. Similarly, in a placebo-controlled study, 13 PD and 34 HD patients were randomized to either oral intake of vitamin E (300 mg daily) or placebo for a period of 20 weeks. Treatment with vitamin E significantly suppressed OS status in both HD and PD patients [54]. In agreement with these results, another study in PD subjects demonstrated that oral intake of the combination of vitamins E and C inhibited formation of prooxidants in urine, blood, and peritoneal fluid [55]. Three open-label trials in maintenance HD patients also reported a beneficial antioxidant effect of oral intake of vitamin E at a dose of 500-800 mg/day [50, 52, 56]. A meta-analysis of 46 randomized controlled trials showed that among various nutritional interventions, only omega-3 fatty acids and vitamin E resulted in a significant decrease in circulating inflammatory markers [57].

In disagreement with these findings, at least six studies reported no effect of vitamin E on OS, while another one surprisingly showed that a-tocopherol triggered oxidation in HD subjects. After oral daily supplementation of 400-800 IU for 2-6 months in cohorts of maintenance HD patients, OS serum biomarkers remained unchanged [43, 48, 58–61]. Antoniadi et al. reported that compared to the placebo group, HD patients treated with oral intake of a-tocopherol 500 mg/day for a year had significantly lower serum levels of antioxidants and therefore were more prone to oxidation [62]. The authors speculated that vitamin E might act as a prooxidant instead of as an antioxidant in certain conditions. Such a condition is often encountered in HD patients, when an adequate amount of vitamin E is combined with severe depletion of other antioxidants which is crucial for the reduction of vitamin E, such as vitamin C.

A systematic review found 56 clinical trials examining the effects of several antioxidant treatments on biomarkers of OS in HD patients. A-tocopherol was by far the most investigated antioxidant agent, with the majority of the studies (20/25) supporting that its administration reduced OS [63]. Furthermore, the SPACE trial, a randomized placebo-controlled study in 196 HD patients with preexisting CVD, showed that compared to placebo, high-dose a-tocopherol supplementation (800 IU/day) for a long period (519 days) reduced the risk of a major CV event (defined as myocardial infarction, angina, peripheral arterial disease, or stroke) [64]. Although several observational studies support vitamin E intake for prevention of OS and CV events, a recent meta-analysis of 135,967 subjects in 19 studies showed that a high dosage of vitamin E (>400 IU/day) was associated with an increased risk for all-cause mortality and therefore should be avoided [65].

#### 3.1.5. Vitamin E-Coated Membranes

Since the biocompatibility of the HD dialyzer triggers formation of ROS during HD sessions, use of vitamin E-coated membranes (VECM) might normalize serum vitamin E levels, improve dialyzer biocompatibility, and subsequently suppress OS [66]. Usberti et al. showed that compared to dialysis with conventional membranes, treatment with VECM was accompanied not only by higher plasma a-tocopherol levels, decreased total thiols, and reduced lipid peroxidation status but also by a significantly better degree of anemia correction [67]. In another study, 43 maintenance HD patients were transferred from high-flux synthetic dialyzers to VECM polysulfone dialyzers. After a 3-month dialysis on VECM, the antioxidant capacity of RBCs was significantly improved (assessed by increased levels of vitamin E and increased SOD activity in RBCs), accumulation of AOPPs was significantly abrogated, and EPO resistance index was reduced [68]. Similarly, a recent study by Locatelli et al. found that in a cohort of 93 chronic HD patients, treatment with VECM polysulfone dialyzers improved the EPO resistance index significantly, compared to treatment with low-flux synthetic membranes [69]. According to these studies, in HD patients, RBC survival and EPO responsiveness are directly influenced and improved by high vitamin E plasma levels and a more effective management of anemia could be achieved by increasing vitamin E levels.

Sosa et al. conducted a systematic review and meta-analysis including 14 trials including 158 HD patients and reported that conversion of HD patients to VECM is accompanied by improvement of the lipid peroxidation status [70]. Another meta-analysis of 15 randomized controlled studies and 503 stable HD subjects demonstrated that VECM significantly decreased circulating plasma levels of c-reactive protein (CRP), interleukin-6 (IL-6), thiobarbituric acid-reactive substances (TBARS), and oxidized-LDL (ox-LDL), without affecting dialysis adequacy [71]. Moreover, in a recent meta-analysis of 60 trials with 2118 maintenance HD patients, VECM was reported to improve erythropoietin resistance index and inhibit oxidation of lipids and inflammation status [72]. Treatment with VECM seems to ameliorate lipid peroxidation status and improve inflammation, anticoagulation, and anemia. Among these beneficial effects of VECM, better management of renal anemia is supported by the most therapeutically relevant and convincing data [73]. There is a growing body of evidence suggesting that in HD patients, the use of VECM is well-tolerated, with no serious adverse effects, and may exert beneficial antioxidant effects.

### 3.2. Vitamin C (Ascorbic Acid)

During HD therapy, a significant amount of vitamin C is lost and also ascorbic acid free radicals are formed contributing thus to enhanced OS. Clermont et al. found that a dialysis session with a highly biocompatible synthetic membrane was accompanied by a significant decrease in plasma vitamin C levels and formation of ascorbyl free radicals. Moreover, when the conventional synthetic dialyzers were replaced with VECM, vitamin C levels were increased and ascorbyl radicals were significantly decreased [21]. Since deficiency of the powerful antioxidant vitamin C is frequent in dialysis patients, several investigators hypothesized that its supplementation might suppress OS in HD patients.

Compared to placebo, daily oral administration of vitamin C (250 mg) significantly decreased plasma OS biomarkers in cohorts of stable HD patients [74, 75], while only a single high dose of vitamin C (2 g) successfully suppressed OS induced by the HD procedure [76]. Moreover, the combined therapy with daily oral vitamins E (600 mg) and C (200 mg) attenuated lipid peroxidation and subsequently improved microcirculation in a cohort of maintenance HD patients [77]. Intravenous administration of vitamin C (1 g) and use of dialysate enriched with ascorbic acid during HD sessions inhibited formation and accumulation of free radicals [17, 78], reduced serum levels of lipid peroxides and AGEs [79], and suppressed DNA oxidation in lymphocytes [80]. In disagreement with these results, several investigators reported that vitamin C failed to attenuate OS, while two studies reported enhanced oxidative activity after vitamin C supplementation. Four trials in maintenance HD patients reported that although either oral or intravenous administration of vitamin C normalized serum vitamin C deficiency, OS status remained unchanged [81–84]. Regardless of the route of administration, both oral (1.5 g) and intravenous (480 mg per HD session) vitamin C triggered oxidative response in chronic HD patients [85, 86].

Although vitamin C deficiency is well-established in PD patients [87, 88] and accompanied by enhanced inflammation [89], only a few investigators examined the possible antioxidant effect of vitamin C supplementation in these patients. Sundl et al. showed that vitamin C levels are strongly and independently correlated with OS status in PD patients and low-dose intake of ascorbic acid might be beneficial [88]. Boudouris et al. reported that oral supplementation of a combination of vitamins C and E in 20 PD patients resulted in significant inhibition of MDA and carbonyl formation and increased TAC [55]. Moreover, vitamin C was strongly and independently associated with hemoglobin levels in a cohort of 56 stable PD patients suggesting that its daily intake might improve erythropoiesis in these patients [90].

As already mentioned, Coombes and Fassett conducted a systematic review including 56 clinical trials examining the antioxidant therapies for OS amelioration in HD patients. The data on vitamin C was controversial; vitamin C was found to reduce OS only in 4 of the 11 studies, increase in 3, and have no effect in 4 other studies, while one trial showed no impact of vitamin C treatment in mortality [63]. The authors of the study hypothesized that the different doses, duration, and routes of administration might explain the heterogeneity of findings. Although vitamin C deficiency is frequent in dialysis patients, based on the existing data, vitamin C supplementation for OS suppression in dialysis patients cannot be recommended.

### 3.3. Vitamins B and Folic Acid

Vitamin B family refers to a group of similar essential nutrients. Vitamin B1 (thiamin), B6 (pyridoxine), B12 (cobalamin), and B9 (folic acid) are the most active compounds of the group. Folic acid belongs to the vitamin B complex and is essential for DNA synthesis and amino acid metabolism. In both the general population and CKD patients, folic acid along with vitamins B6 and B12 are a well-established treatment for reducing serum levels of homocysteine, a biomarker of protein oxidation linked with CVD [91, 92].

*In vitro*, supplementation with folic acid and B12 significantly attenuates the genomic damage in chronic HD patients' lymphocytes [93]. A growing body of evidence suggests that B6 is an effective inhibitor of advanced glycation and lipoperoxidation processes [94–96], directly scavenging free radicals formed during lipid oxidation and neutralizing them. In diabetic rats, supplementation with pyridoxamine improved albuminuria and prevented deterioration of renal function [97].

Two multicenter placebo-controlled trials in patients with established diabetic nephropathy showed that compared to placebo, a daily oral intake of pyridoxamine (doses ranged from 100 to 500 mg) for 6 months resulted in significant preservation of renal function and decreased urinary levels of inflammatory cytokines [98]. Bayes et al. enrolled 16 chronic HD patients who received 400 mg of the well-known antioxidant vitamin E for 3 months per OS daily. After a 4-week washout period, all patients were treated with intravenous 10 mg of folic acid, thrice weekly after dialysis sessions for another 3 months. The authors found that compared to vitamin E, folic acid significantly decreased serum homocysteine (-44%) and MDA levels (-40%) (a biomarker of lipid peroxidation) and therefore might reduce the risk of atherosclerosis [19]. In agreement with these results, a multicenter, longitudinal, open intervention trial in 32 maintenance HD patients showed that compared to baseline, three-month treatment with low dose of folic acid (1 mg intravenously, thrice weekly after every HD session) resulted in a significant decrease in serum homocysteine and MDA levels [99]. A randomized, double-blind, placebo-controlled trial including 46 chronic HD patients investigated the effect of supplementation of folic acid (oral 10 mg, thrice weekly after dialysis session) or placebo for 6 months on total antioxidant capacity (TAC), homocysteine, and hydroperoxide plasma levels [100]. Folic acid treatment successfully reduced serum homocysteine levels and increased TAC in this study and therefore might ameliorate CV risk. On the other hand, Nascimento et al. conducted a multicenter, randomized, double-blind, placebo-controlled study on 50 stable HD patients to investigate the possible effect of B6 and B1 intake on OS. Patients were randomly assigned to a combination of high doses of pyridoxine (200 mg) and thiamine (250 mg) or placebo for 2 months. After the study period, both groups had similar plasma levels of OS and inflammation biomarkers [101].

Although combination therapy with folic acid and B6/B12 is considered a favorable treatment for homocysteinemia in HD subjects, a recent meta-analysis of 6 studies and 2452 dialysis patients showed that homocysteine-lowering therapy (including folic acid, B6, and B12 supplementation) had little or no effect on CV events and all-cause and CV mortality in these patients [102].

### 3.4. Vitamin D Analogues

Dialysis patients with secondary hyperparathyroidism (SHP) exhibit accelerated OS and inflammation [103]. Wu et al. conducted a study to assess the effect of vitamin D analogues—currently a first-line treatment for SHP—on the oxidation and inflammatory status in 25 stable HD patients. The authors demonstrated that, compared to baseline, after a 16-week treatment with calcitriol, several serum inflammatory and OS biomarkers were significantly suppressed [103]. Similarly, Tanaka et al. showed that intravenous calcitriol not only attenuated accumulation of free radicals but also strengthened endogenous antioxidant activity in HD subjects with SHP [104]. In a similar group of patients, serum levels of several OS and inflammatory biomarkers were markedly decreased, while activity of the circulating antioxidants CAT, SOD, and GSH was significantly upregulated after a 3-month intravenous treatment with selective vitamin D receptor activators [105]. A randomized, controlled, double-blind study enrolled 60 diabetic HD patients, randomly divided to receive 50,000 IU vitamin D3 or placebo every 2 weeks for 12 weeks. Compared to placebo, those treated with vitamin D exhibited significantly reduced plasma levels of MDA and high-sensitivity CRP (hsCRP) and higher TAC [106].

In PD patients, the data regarding the effect of vitamin D analogues on OS are scarce and only derived from experimental studies. *In vitro* as well as *in vivo* studies in mice showed that the active form of vitamin D inhibited accumulation of free radicals and glycoxidation and therefore might preserve the peritoneum homeostasis, through amelioration of OS status [107–109].

One of the pleotropic effects of vitamin D in dialysis patients might be amelioration of OS status. Currently, the data on this are extremely limited and therefore its supplementation should not be based on its antioxidant properties.

### 3.5. Coenzyme Q10

Coenzyme Q10 is a vitamin-like fat-soluble element, essential for energy metabolism. The liver, heart, and kidneys have increased energy requirements and therefore have the highest coenzyme Q10 endogenous levels [110]. Coenzyme Q10 is an electron carrier and therefore might act as an ROS scavenger. Q10 supplementation suppressed PMN activation and therefore decreased the OS status in a cohort of 11 kidney transplant recipients. Studies in animal models with hypertension and diabetes showed that coenzyme Q10 improved hypertension, protected the kidney from damage [111, 112], reversed OS biomarkers in the liver, heart, and kidneys [113], and neutralized the accumulating free radicals resulting from drug nephrotoxicity [114, 115].

Sanaka et al. investigated the effect of coenzyme Q10 supplementation on the OS status of 36 stable HD patients for 6 months and found that although the plasma concentrations of oxidative marker MDA and advanced oxidation protein products (AOPPs) were significantly reduced, serum indicators of antioxidant status were also paradoxically decreased [116]. To examine the safety and efficacy of several doses of Q10 administration in HD patients, Yeung et al. conducted a dose escalation study in 15 maintenance HD patients and reported that daily, oral Q10 supplementation at doses as high as 1800 mg was well-tolerated and significantly reduced serum F2-isoprostanes, in a dose-dependent manner [117]. In disagreement with these results, a randomized, placebo-controlled trial including 23 stable HD patients failed to show any significant beneficial effect of daily low dose of Q10 (200 mg) on exercise performance and OS biomarkers, compared to placebo [118]. Rivara et al. conducted a recent randomized, double-blind, placebo-controlled trial in 65 maintenance HD patients, randomly divided to 3 groups: oral daily supplementation with 600 mg or 1200 mg Q10 or matching placebo for 16 weeks. Compared to placebo and low-dose, only 1200 mg of Q10 was accompanied by a significant reduction in serum F2-isoprostane levels [119]. Based on the recent published data, a safe and efficient antioxidant dose of Q10 in HD patients could be 1200–1800 mg per day, but its beneficial effects remain to be further confirmed.

### 3.6. L-Carnitine

L-Carnitine is an amino acid-derived compound crucial to energy metabolism. Additional to its antioxidant activity, L-carnitine may play a pivotal role in preventing deterioration of renal function in CKD patients [120]. Moreover, carnitine supplementation in HD patients improved several indices including renal anemia, fatigue, cardiac function, physical function, and quality of life [120–122]. Although at first, carnitine therapy was considered to improve anemia status [123], a more recent meta-analysis of 49 trials failed to show any effect of carnitine supplementation on hemoglobin levels and EPO dose in 1734 HD patients [124]. In 2014, a meta-analysis of 25 randomized, placebo-controlled trials including 1172 maintenance HD patients failed to show any beneficial effect of carnitine intake on anemia, dyslipidemia, SHP, nutritional, inflammation, and OS status [125]. However, another meta-analysis also published in 2014, including 49 randomized controlled trials with 1734 stable HD patients, reported that carnitine supplementation significantly decreased plasma LDL and CRP levels and therefore might protect against CV events in this group of patients [124]. The disagreement of the two meta-analyses could be attributed to the heterogeneity and different designs of the studies included. In dialysis patients, carnitine supplementation as an antioxidant and anti-inflammatory supplement cannot be justified in routine clinical practice.

### 3.7. Statins/Omega-3 Polyunsaturated Fatty Acids

Omega-3 fatty acids are long-chain polyunsaturated fatty acids, obtained through diet. The most bioactive types of omega-3 are eicosapentaenoic acid (EPA) and docosahexaenoic acid (DHA). Due to their structure, omega-3 fatty acids are susceptible to oxidation, and since dialysis patients are prone to lipid peroxidation and atherosclerosis, supplementation with omega-3 in dialysis may protect from CV events [126]. Besides their anti-inflammatory properties, omega-3 fatty acids have been suggested to exert antioxidant effects, through upregulation of the GSH antioxidant system. Administration of EPA and DHA reduced renal fibrosis, inflammation, and OS status in rats with obstructive renal disease [127] and significantly suppressed inflammation and endothelial damage in HD patients [128]. Two randomized placebo-controlled trials in chronic HD patients highlighted the beneficial effect of EPA intake on several oxidative and inflammatory markers [129, 130], while another recent study with a similar design reported that daily EPA supplementation had no effect on OS status, in a cohort of chronic HD patients [131]. Intake of omega-3 fatty acids in HD patients has been shown to improve nutritional, inflammatory, and anemia status and subsequently OS [132, 133]. A very recent meta-analysis of 46 randomized controlled trials demonstrated that among various nutritional interventions, only omega-3 fatty acids and vitamin E reduced significantly serum CRP levels [57].

There is limited data on the effects of omega-3 acid administration in PD patients. A randomized, placebo-controlled, double-blind study included 90 ESRD patients on chronic ambulatory PD (CAPD), randomized to placebo or oral daily treatment with 3 g of omega-3 for 8 weeks. After the study period, treatment with omega-3 failed to show any change in lipid parameters, erythropoiesis status, and OS status assessed by serum levels of antioxidants SOD and GSH [134]. Two other randomized, controlled, double-blind trials on CAPD patients showed that compared to placebo, oral treatment with omega-3 for 2 months did not affect the lipid profile or inflammatory markers [135, 136].

Moreover, two recent meta-analyses, one including 710 studies and 7,917 patients with history of coronary heart disease -and therefore with heavy CV burden similar to ESRD patients- and another including 79 trials and 112,059 subjects from the general population generated the same result; omega-3 fatty acid supplement failed to show any protective effect on all-cause mortality, CV mortality, and CV events in both cohorts [137, 138].

Statins are lipid-lowering drugs, inhibiting directly the HMG-CoA reductase enzyme. In addition to prevention of CV events, several investigators have suggested that statin use might ameliorate OS in dialysis patients. The 4D study showed that compared to the placebo group, stable HD patients that were treated with atorvastatin 40 mg/day for 3 months had a mean 36.5% reduction of ox-LDL plasma levels [58]. Likewise, a cohort of 103 maintenance HD patients was randomized to either statin treatment or no treatment for 6 months; statin use significantly increased the serum levels of selenium, an antioxidant trace element [139]. In a similar patient cohort, even a low daily intake of simvastatin (5 mg for 6 months) can prevent the formation of lipid peroxides in [140]. Likewise, another study showed that treatment with simvastatin for just one week improved endothelial dysfunction and ameliorated OS status assessed by ox-LDL and isoprostane serum levels in HD patients [141].

A meta-analysis of 14 studies and 2086 dialysis patients (HD and PD) showed that compared to placebo, statins failed to decrease all-cause and CV mortality, although one single study in 1255 HD patients reported a lower incidence of nonfatal CV events with statins compared to placebo [142]. Another meta-analysis included 9 studies and 3098 HD patients and highlighted that statins can successfully suppress the chronic, systemic inflammation that accompanies ESRD and therefore might ameliorate inflammation-derived OS [143].

The data regarding statin and omega-3 supplementation as antioxidants in dialysis patients are controversial and limited, and therefore, the current evidence cannot justify their administration. Additional studies on large cohorts are needed in order to draw a definite conclusion.

### 3.8. A-Lipoic Acid

A-Lipoic acid (ALA) is a mitochondrial element with an important role in energy metabolism. ALA is a natural inhibitor of cell oxidation by directly scavenging ROS, chelating trace elements and iron ions, and recycling endogenous antioxidant enzymes and vitamins including Q10, GSH, and vitamins E and C [144–146]. Moreover, ALA is involved in lipid and glucose metabolism [146] and inhibits vascular calcification [147]. *In vitro* and *in vivo* studies showed that acute kidney injury (AKI) caused by ischemia/reperfusion could be significantly ameliorated by ALA [148, 149]. Experimental animal models with diabetic nephropathy showed that ALA prevented renal injury and glomerulosclerosis by decreasing MDA, improving glycemic control, and increasing GSH levels [150–152]. Several investigators highlighted the renoprotective effect of ALA on adriamycin-induced nephrotoxicity, a state of enhanced OS [153, 154], cyclosporin nephrotoxicity [155, 156], and toxic doses of acetaminophen [157]. These studies suggested that ALA could have a therapeutic result in cases where tissue injury is a result of free radicals and therefore human studies in dialysis patients were initiated.

The data on the effect of ALA supplementation on OS, inflammation, and erythropoiesis in dialysis patients remains however controversial. Khabbazi et al. randomized 63 maintenance HD patients to oral daily intake of 600 mg ALA or placebo for 8 weeks and found that although ALA intake caused a significant decrease in hsCRP serum levels, there was no effect on TAC and MDA concentrations [158]. Safa et al. reported no effect of ALA administration on IL-8 and tumor necrosis factor (TNF-a) serum levels of HD patients [159]. In disagreement with these results, Chang et al. randomized 50 chronic HD patients to ALA supplementation (600 mg/day for 12 weeks) or no treatment and showed that ALA intake caused a significant decrease in serum asymmetric dimethylarginine (ADMA), a well-known biomarker of OS and independent risk factor for CVD in these populations [160]. In another trial, 44 HD patients treated with EPO were divided in two groups: those treated with 600 mg ALA oral daily for 3 months and the control group. Although MDA, ADMA, ox-LDL, IL-6, and TNF-a plasma levels were similar in both groups at baseline and at the end of the study, EPO doses and the EPO resistance index were significantly reduced in the ALA group, but remained unchanged in the control group [161]. In a randomized controlled, double-blind study, a combination of ALA (600 mg/day) and vitamin E (400 IU) for 8 weeks improved nutritional and inflammatory status but had no effect on lipid peroxidation status of 85 chronic, HD subjects [162]. Himmelfarb et al. carried out a randomized, placebo-controlled, double-blind trial to examine the effects of antioxidant therapy on anemia, inflammation, OS, mortality, and morbidity in a large cohort of 353 stable, chronic HD patients [163]. All patients were randomized to receive either combination of ALA (600 mg/d) and mixed a and *γ* tocopherols (666 IU/d) or placebo for 6 months. Antioxidant therapy had no effect on circulating inflammatory biomarkers (hsCRP, IL-6), serum levels of F2-isoprostanes, isofurans, and anemia status. Mortality and hospitalization rates were similar in both groups. The authors concluded that although administration of mixed tocopherols with ALA was well-tolerated with no major side effects, it failed to show any beneficial effect in this group of patients.

### 3.9. Trace Elements

Trace elements are compounds with very low concentrations (<50 mg/kg of body weight) in the human body but with very important functions. Zinc (Zn), copper (Cu), and selenium (Se) are micronutrients playing a pivotal role in biological systems. Zn is an essential element for enzymes and proteins, including the natural endogenous antioxidant SOD. Cu is used for hemoglobin synthesis and bone metabolism and is present in SOD. Se is important for thyroidal metabolism, growth of the human body, and fertility. It is a natural inhibitor of lipid oxidation and an important component of the endogenous antioxidant glutathione peroxidase (GPx). Moreover, in HD patients Se deficiency has been associated with risk for all-cause mortality [164, 165], hospitalization [165], and accelerated OS status [2]. Plasma levels of trace elements might be altered in both HD and PD patients [166]. There is a growing body of evidence suggesting that trace elements and especially selenium levels in plasma, RBCs, and WBCs are low in HD and PD patients compared to healthy controls [164, 167–171]. Healthy controls had higher serum Se levels compared to PD patients, and HD patients had even lower levels than PD counterparts [167, 170].

In a cohort of HD patients, randomly assigned to either Zn intake (oral 220 mg/day) or placebo for 42 days, Zn supplementation led to a significant reduction in CRP serum levels [172]. In agreement with these results, two double-blind, placebo-controlled, randomized studies in 60 HD patients showed that compared to placebo, 2-month supplementation with Zn (100 mg/day per OS) resulted in significant upregulation of antioxidant enzymes [173], increased antioxidant status (assessed by a significant increase of serum TAC, GSH, and SOD), and improved lipid peroxidation status by decreasing plasma MDA levels [174]. Moreover, Zn supplementation in dialysis patients (HD and PD) improved Se serum levels and ameliorated OS status [175]. A recent meta-analysis of 15 randomized controlled studies examined the effects of Zn intake on HD patients and demonstrated that Zn supplementation was strongly and independently associated with reduced serum CRP levels, higher SOD plasma levels, improved nutrition status, and lower plasma MDA concentrations [176]. Zn beneficial effects presented a time-effect relationship.

In a cohort of stable HD patients, Se supplementation resulted in upregulation of RBC GPx activity compared to placebo, although plasma GPx remained unchanged [177]. The authors of this study concluded that in dialysis patients, the damaged kidney loses the ability to synthesize GPx, even after Se supplementation. In HD patients, Se intake (200 *μ*g per OS daily) for 3 months prevented oxidation of DNA in leukocytes [178], improved nutrition and inflammation status, and reduced MDA serum levels [179]. Furthermore, combined supplementation of 600 *μ*g Se with 400 IU vitamin E before the HD session attenuated the iron infusion-induced MDA accumulation in the circulation [180].

Although all trace elements are deficient in dialysis patients, only Se plasma levels have been directly and inversely linked with mortality. Further studies, in larger cohorts, with longer periods of treatment could be useful for providing more evidence regarding the clinical utility of Se intake.

### 3.10. Curcumin

Curcumin is a polyphenol, active element of the herbal spice *Curcuma longa* (or turmeric) with surprisingly pleiotropic beneficial properties, including anti-inflammatory and antioxidant activity. Due to its complex chemical structure, curcumin has the ability to scavenge free radicals directly by donating hydrogen ions and therefore neutralizing them and upregulate indirectly the expression of the antioxidant enzymes SOD, CAT, and GSH [181]. *In vitro* studies showed that preincubation of activated macrophages of rat peritoneum with curcumin inhibited the accumulation of ROS by macrophages [182] and might be a stronger inhibitor of lipid peroxidation compared to a-tocopherol [183]. Similarly, intraperitoneal injection of curcumin in rats with endotoxin-induced peritonitis ameliorated protein and lipid oxidation [184]. Several *in vivo* studies reported that daily supplementation of curcumin (15-150 mg/kg) for 2 to 8 weeks in rats preserved kidney function and inhibited macrophage activation through upregulation of antioxidant enzymes and suppression of inflammatory cytokines [185–188]. Similarly, in animal models with severe CKD or AKI, curcumin administration markedly preserved renal function, suppressed OS, and ameliorated albuminuria and inflammation [189–197].

The data regarding the antioxidant and anti-inflammatory effects of curcumin administration in HD patients is limited but quite promising. Shoskes et al. randomized 43 HD-dependent kidney transplant recipients to 3 groups: low dose of 480 mg/day curcumin per OS, high dose of 960 mg, or placebo. The study period started after transplant surgery for a period of one month. Treatment with a high dose of curcumin was associated with early graft function and prevention of acute graft rejection, probably through suppression of OS and inflammation [198]. Another randomized, double-blind study included 50 stable HD patients, randomly divided to either treatment with curcumin (1500 mg/day per OS) or placebo for 2 months. Compared to placebo, therapy with curcumin resulted in significant reduction in serum MDA levels and upregulation of the antioxidant CAT activity and concentration in erythrocytes [199]. A double-blind, placebo-controlled randomized study with a very similar design showed that compared to placebo, daily oral administration of 500 mg of curcumin for 2 months was accompanied by significant decrease in plasma MDA levels and increase in antioxidants GPx, and CAT in RBCs, in a cohort of 45 maintenance HD patients [200]. Besides its antioxidant properties, a recent trial in HD patients showed that oral supplementation with curcumin in HD patients suppressed chronic systemic inflammation (serum levels of IL-6, hsCP) and subsequently might protect against inflammation-derived OS in these patients [201].

### 3.11. Polyphenols

Resveratrol (3,5,4-trihydroxystilbene) is a plant polyphenolic compound found in grapes, red wine, and berries. Resveratrol is a direct free radical scavenger and indirectly upregulates expression of the endogenous antioxidant genes and enzymes SOD, CAT, and GPx [202]. In animal models of ischemia/reperfusion AKI [203, 204], septic AKI [205, 206], gentamycin [207], cisplatin [208], and cyclosporin nephrotoxicity [209], as well as diabetic nephropathy [210], resveratrol, due to its anti-inflammatory and antioxidant properties, attenuated OS-mediated kidney damage.

In humans, Castilla et al. randomly divided 32 maintenance HD patients to groups of dietary supplementation with red grape juice, vitamin E, both, or placebo for 2 weeks. In this cohort of patients, red grape juice, a source of polyphenols, decreased oxidative activation of PMNs, serum ox-LDL levels, and circulating inflammatory biomarkers to a greater extent compared to vitamin E and placebo [211]. The authors concluded that polyphenols might reduce CV risk in HD patients. To investigate the possible antioxidant beneficial effect of polyphenols in HD patients, the same group of investigators randomly assigned 38 HD patients to either daily consumption of 100 mL red grape juice for 2 weeks or nothing. Polyphenol juice intake led to a significant increase in TAC, decrease in serum ox-LDL levels, and improved lipid profile parameters [212]. Spormann et al. enrolled 21 HD patients to consume daily 200 mL of polyphenolic-rich red fruit juice (21 days run-in, 30 days juice intake, and 21 days washout period) and found that protein, lipid, and DNA oxidation were significantly decreased and glutathione serum levels were increased during juice consumption [213]. Wu et al. randomized 33 HD patients to daily per OS intake of 100 mg purified pomegranate polyphenol extract or placebo for 6 months and demonstrated that compared to the placebo group, polyphenol treatment decreased blood pressure and increased antioxidant activity [214]. In agreement with these results, another recent study reported that 6-month supplementation of unfermented grape juice, rich in polyphenols, decreased the levels of DNA oxidation in a cohort of HD patients [215]. Moreover, in PD patients, high-dose supplementation with resveratrol improved ultrafiltration by attenuating angiogenesis and OS derived by use of PD fluids [216]. A recent meta-analysis of 12 studies evaluated the effect of polyphenol-rich supplementation (including grape, pomegranate, and turmeric) on CV risk in patients undergoing maintenance HD. Although polyphenol-rich interventions significantly decreased blood pressure and serum triglycerides and MPO (a biomarker of protein oxidation status) levels, the studies included had unclear risk of bias and provided low evidence.

### 3.12. Green Tea

Green tea is composed of vitamins, minerals, and catechins-well known and effective free radical scavengers. Besides its antioxidant properties, green tea has been suggested to protect against atheromatosis and CVD, in the general population as well as CKD and dialysis patients [217–219]. A meta-analysis of 31 studies found low-grade evidence that chronic, daily, consumption of green tea at 600-1500 mL decreases lipid oxidation, enhances TAC, and subsequently might protect against CVD. These beneficial effects of green tea were more profound in patients exposed to high OS [220], and since CKD and dialysis are states of accelerated oxidation, it seemed legitimate to hypothesize that green tea might be beneficial for these patients.

Several studies in animal models with various types of renal damage (diabetic nephropathy, lupus nephritis, glomerulonephritis, and urethral obstruction) suggested that green tea treatment might protect against protein and lipid oxidation, prevent atherosclerosis, and preserve renal function [221–226].

Human studies in dialysis patients consuming green tea are scarce. Park et al. divided 40 chronic dialysis patients to two groups: those that consumed green tea at a high dose (5 g/day) and those who consumed equal amounts of water for 4 weeks and found that green tea intake improved significantly the endothelial function through suppression of OS and inflammation [218]. Similarly, another study in 44 HD patients showed that compared to placebo, those treated with 455 mg/day of catechins (green teat extract) were benefitted by significant reduction of plasma proinflammatory, prooxidant, and atherogenic molecules [219]. In the same cohort, catechins were reported as more efficient antioxidant scavengers than vitamin C. In agreement with these results, Calo et al. reported that 6-month supplementation with green tea (1 g/day) in 20 stable, chronic HD patients led to a significant decrease in OS and inflammatory state and improvement of cardiac function (assessed by a decrease in left ventricular mass). The authors of the study concluded that green tea, besides its anti-inflammatory and antioxidant properties, might also exert an antiatherogenic protective effect in these high-risk patients [227].

There is some evidence that green tea consumption might offer protection from atherosclerosis and CVD and ameliorate OS in dialysis patients. The data however is extremely limited and insufficient to draw any definite conclusion. Since there are no serious adverse effects, further large cohort studies are warranted in order to establish its antioxidant and antiatherogenic effect in dialysis patients.

### 3.13. Flavonoids

Flavonoids are natural compounds with pleiotropic properties and potential therapeutic applications. Silibinin is a flavonolignan bioactive ingredient of silymarin, a flavonoid extracted from the milk thistle plant. Due to its anti-inflammatory and antifibrotic effects, silymarin is a traditionally used natural supplement for liver and kidney conditions. For over 20 years, silymarin has been successfully used for prevention and treatment of mushroom poising-derived AKI in both animals [228, 229] and humans [230]. Adriamycin and cisplatin are powerful chemotherapeutic drugs with important side effects such as kidney injury (owing to severe acute oxidative and inflammatory cell damage). Several investigators suggested that silymarin may prevent renal injury caused by these drugs through antioxidant and anti-inflammatory processes [231–233]. Since mushroom poising, cisplatin, and adriamycin cause nephrotoxicity through enhancing OS, it was reasonable to hypothesize that silymarin might protect against accumulation of free radicals in CKD. Silymarin is a natural free scavenger of ROS that inhibits oxidation of lipid cell membranes [234]. *In vitro*, silymarin not only prevented oxidative damage in renal cells incubated with high glucose concentrations [235] but also stimulated biosynthesis of DNA and protein in monkey renal cells [236]. Tager et al. treated peritoneal macrophages from peritoneal effluent of 30 stable, chronic CAPD subjects with silibinin for 35 days and found that flavonoid treatment ameliorated thiol oxidation and restored peritoneal cell functional abilities [237]. In a similar *in vitro* study, Dietzmann et al. measured thiol status and TNF-a release of blood lymphocytes from healthy controls and 30 maintenance HD patients with diabetes. Compared to controls, HD cells presented a significant thiol deficiency which correlated directly to elevated production of TNF-a. Compared to untreated dialysis lymphocytes, treatment of blood white cells with 350 mg silibinin resulted in restoration of thiol status in vitro and in vivo, accompanied with reduction in TNF-a production [238].

In animal models, although silymarin in rat renal cells failed to decrease DNA and lipid oxidation caused by glycerol [239], other investigators demonstrated that pretreating rats with silymarin can lessen or prevent structural and functional injury of kidney cells caused by ischemia/reperfusion [240, 241].

A randomized, controlled, double-blind study in 60 patients with diabetic kidney disease showed that compared to placebo, daily therapy with 420 mg silymarin resulted in a significant decrease in albuminuria and reduction in urinary levels of TNF-a and MDA [242]. Therefore, the authors suggested that silymarin might be beneficial for preventing albuminuria and progression of diabetic nephropathy, through suppression of inflammatory and oxidative processes. Roozbeh et al. randomized 80 HD patients to 4 groups: 420 mg/day silymarin per OS, 400 IU/day vitamin E, combination of silymarin and vitamin E, or placebo for 3 weeks. Compared to the other groups, combination of vitamin E and silymarin led to a decrease in serum MDA levels, increase in RBC levels of the antioxidant GPx, and increase in hemoglobin levels [243]. Nazemian et al. treated 15 chronic PD patients with 210 mg/day silymarin for 8 weeks and demonstrated that silymarin supplementation significantly elevated hemoglobin levels and decreases serum TNF-a concentrations [244]. The authors of this study concluded that silymarin might be useful in the treatment of OS and inflammation for PD patients. In a recent study, 50 PD patients were randomized to 420 mg/day of silymarin or placebo for 2 months and OS biomarkers were measured in plasma and erythrocytes at baseline and after the end of the study period. Compared to placebo, silymarin significantly increased the activity of the antioxidant endogenous enzyme CAT in RBCs and improved anemia status (assessed by an increase of hemoglobin levels) [245].

Studies with larger cohorts, hard end-points, and longer treatment periods are required to elucidate the effect of flavonoids on CV outcomes and OS in dialysis patients.

### 3.14. N-Acetylcysteine (NAC)

Due to impaired thiol renal metabolism and imbalance of circulating redox pairs (such as cysteine/cystine and reduced/oxidized glutathione), a defective homeostasis of blood thiols accompanies CKD and is further exacerbated by HD treatment [246]. Khazim et al. conducted a cross-sectional study in 33 maintenance HD patients and 21 healthy controls and found that compared to controls, the RBC GSH/GSSG redox potential was significantly lower and both glutathionylated and cysteinylated hemoglobins were significantly higher in HD subjects. Moreover, these thiols were strongly associated with measures of uremia [247]. In a population of 98 maintenance HD patients, Galli et al. showed that compared to the standard 4 h thrice weekly schedule, frequent, 2 h daily HD resulted in an improved plasma thiol status [248]. NAC is a water-soluble, thiol-containing antioxidant that directly scavenges circulating free radicals and replenishes glutathione stores [249]. NAC has been repeatedly demonstrated to protect against contrast-induced nephropathy, through suppression of the OS-derived by renal tubular injury [250]. *In vitro*, NAC not only ameliorated oxidative activation of HD subjects' leukocytes, triggered by accumulating oxidative products (such as AOPPs), but also reversed WBCs' oxidative activation. The authors of these studies suggested that NAC might have a therapeutic clinical use to relief dialysis-induced OS [251, 252]. Recent *in vitro* studies showed that compared to the hydrophilic NAC, the lipophilic precursor form of NAC (NAC ethyl ester) is even more effective in increasing the intracellular concentrations of the antioxidant thiol, GSH. This fact might explain the discrepancy between results of *in vitro*/preclinical trials where NAC was found to be an effective GSH enhancer and clinical studies that failed to show any beneficial effect of NAC [253].

During PD, the accumulating AGEs generated in the peritoneum gradually cause severe injury and damage through glycoxidation and inflammation. Several investigators showed that NAC might ameliorate AGE-related OS and therefore protect the peritoneum from injury [254]. *In vitro*, NAC suppressed formation of N (epsilon)-(carboxymethyl)lysine, an antigen of AGEs involved in the pathogenesis of peritoneal damage found in PD patients [255] and protected human peritoneal mesothelial cells from peritoneal solution-derived injury by preserving the endogenous antioxidant-reduced glutathione and therefore suppressing OS status [256, 257]. Studies on experimental animal models showed that NAC improved ischemia/reperfusion kidney injury [258, 259], attenuated cisplatin nephrotoxicity [260], and inhibited uremia-derived vascular calcification [261]. Moreover, in rat models, NAC was reported to be more beneficial compared to peritoneum resting in preventing peritoneal sclerosis [262] and inhibited the structural and functional ROS-induced damages [263].

The first study of NAC intake in HD patients was carried out by Trimachi and coworkers: compared to no treatment, oral administration of NAC (600 mg twice daily) for 4 weeks resulted in significant reduction in plasma MDA levels [264]. A randomized, controlled, double-blind trial included 40 maintenance HD patients randomly allocated to receive high-dose intravenous NAC (5 g) or placebo. NAC treatment was accompanied by significant reduction in serum ADMA levels, compared to the placebo group [265]. Likewise, Hsu et al. demonstrated that compared to placebo, oral administration of NAC (600 mg per OS daily) not only significantly reduced serum concentrations of 8-isoprostanes and ox-LDL but also improved renal anemia status [266]. Similar results were published by Giannikouris et al.; treatment with NAC reduced circulating levels of ADMA and MDA and improved anemia status in HD patients [267]. Moreover, 1200 mg daily per OS treatment of NAC for 3 months successfully suppressed SHP, chronic inflammation, and OS status in a cohort of maintenance HD patients [268]. A double-blind, randomized, placebo-controlled trial evaluated the effect of NAC on antioxidant defense mechanisms of chronic HD patients and demonstrated that compared to placebo, oral NAC supplementation was accompanied by a significant increase in serum TAC levels [269]. Another study with a similar design randomly allocated 37 maintenance HD patients undergoing kidney transplantation, to receive NAC (per OS 600 mg twice daily) or placebo for 15 days. Compared to the placebo, the NAC group presented significant improvement in GPx activity in RBCs, immediate graft function, and estimated glomerular filtration rate (eGFR) in the first week [270]. The authors concluded that NAC might exert a combination of antioxidant and renoprotective properties in kidney transplant recipients. Several investigators examined the possible effect of NAC on homocysteine serum levels in dialysis patients. Friedman et al. found no effect of 2400 mg of daily per OS treatment with NAC on homocysteine serum levels, compared to placebo in a cohort of 38 HD patients [271], and Bostom et al. failed to show any effect of oral NAC administration (1200 mg) on total plasma homocysteine levels of 11 maintenance HD patients [272]. In disagreement with these results, a randomized, placebo-controlled, crossover trial reported a NAC-dependent rise of homocysteine removal during HD session which resulted in improved endothelial dysfunction [273]. Moreover, intravenous NAC during HD sessions was found to significantly decrease total homocysteine plasma levels in maintenance HD patients [274, 275]. The beneficial effect of NAC on homocysteine removal was more pronounced in dialysis patients with residual renal function (RRF). Furthermore, NAC supplementation not only inhibited the accumulation of prooxidants (serum MDA levels) induced by intravenous iron administration during HD sessions but also fortified the antioxidant defense mechanisms in these patients [276, 277].

Similarly, the data on NAC administration in PD patients for antioxidant protection is promising. Nascimento et al. enrolled 30 chronic, stable PD patients in a placebo-controlled trial and divided them to two age-matched groups: the placebo group and those treated with oral NAC (600 mg twice daily) for 2 months. At baseline and after the study period, OS markers (AOPPs, ADMA, GSH, and homocysteine) and proinflammatory cytokines (hsCRP, IL-6, and TNF-a) were determined in serum. The authors found that, although NAC treatment led to a significant decrease in serum IL-6 levels, all other OS and inflammatory biomarkers remained unaffected. Purwanto and Prasetyo conducted a placebo-controlled trial to examine the possible effect of NAC on inflammation status of CAPD subjects. Thirty-two CAPD patients were allocated to either daily oral supplementation with NAC (600 mg twice daily) or placebo for 2 months. Compared to placebo, NAC administration significantly reduced the levels of several inflammatory biomarkers such as IL-1, IL-6, hsCRP, TNF, and procalcitonin in this group of patients [278]. In dialysis patients, loss of RRF was strongly and independently associated with high risk for CV morbidity and all-cause mortality [279]. Moreover, preservation of RRF was tightly linked with reduction of OS status in these patients [280, 281]. NAC intake might help RRF preservation in both HD and PD patients, possibly through attenuation of OS status. Feldman et al. treated 10 PD patients with oral NAC (1200 mg twice daily for 1 month) and found that compared to baseline, at the end of the study, RRF was significantly improved [282]. In a following study by the same group of investigators, oral supplementation with NAC (1200 mg twice daily for 2 weeks) in a cohort of 20 chronic HD patients with residual urine volume > 100 mL/24 h improved RRF significantly [283]. In agreement with these results, Ahmadi et al. conducted a recent randomized, multicenter, open-label study in 47 maintenance HD patients and found that compared to no treatment, NAC intake (1200 mg twice daily, per OS for 3 months) led to significant improvement of RRF [284].

A systematic review of antioxidant treatment in HD patients included 56 studies and showed that compared to all other agents, NAC was the most efficacious supplement for ameliorating OS [63].

Besides its antioxidant properties, NAC might protect dialysis patients from CVD. In a cohort of HD patients, Sahin et al. suggested that treatment with NAC improved endothelial dysfunction, the first crucial step in the pathogenesis of atherosclerosis [285]. Tepel et al. conducted a randomized, placebo-controlled trial including 134 maintenance HD patients, randomly allocated to receive 600 mg daily oral NAC or placebo for 2 years. Compared to the placebo group, NAC treatment significantly reduced CV events and CV mortality in this group of patients [286].

NAC is an inexpensive, well-tolerated powerful antioxidant with promising results in dialysis patients.

Currently, there are 12 ongoing clinical trials examining the effect of antioxidants on OS in CKD and HD patients: 2 studies on vitamin E (alone or combined with NAC) in HD, 1 trial on VECM treatment of critically ill patients requiring HD, and 1 study on vitamin C in HD subjects. Statin used as an antioxidant in CKD is examined in 2 studies, curcumin is studied in 2 cohorts of CKD and diabetic nephropathy patients, and coenzyme Q10 and resveratrol are examined in similar populations. Moreover, in patients with diabetic kidney disease, green tea consumption and combination of NAC and silibinin are currently tested as potential antioxidants.

## 4. Conclusions

Patients on RRT exhibit excessive OS and subsequently increased risk for CVD and mortality. Both HD and PD trigger the oxidation process, through different pathogenetic mechanisms. Supplementation with several exogenous antioxidants to suppress OS and inflammation has been suggested in these patients. The data regarding the beneficial antioxidant effect of omega-3 fatty acids, statins, coenzyme Q10, curcumin, trace elements, vitamins B and D, green tea, flavonoids, and polyphenols remain controversial; NAC and a-tocopherol seem to have the most promising results in dialysis patients. However, currently none of these compounds are recommended by PD or HD guidelines. Further studies in large cohorts of dialysis patients are required, to establish causality between antioxidant supplementation and clinical hard end-points of CVD and all-cause mortality.

## Figures and Tables

**Table 1:** Possible biomarkers of oxidative stress (oxidants and antioxidants) in hemodialysis.

Lipid oxidation	Free radicals	Protein oxidation	DNA oxidation	Antioxidants
TBARS MDA Ox-LDL F2-isoprostanes Lipoperoxides PCOOH	Nitric oxide ROS	Oxidized albumin MPO ADMA AOPPs AGEs Homocysteine Thiols Carbonyls	8-OHdG 8-OxodG	*Endogenous* TAC SOD CAT GSH GSH-Px GST GSSG GSH/GSSG Ox-LDL antibodies Uric acid Albumin Transferrin, ferritin *Exogenous* Zn, Cu, Se NAC Vitamin B complex Vitamin E (a-tocopherol) Vitamin C (ascorbic acid) Polyphenols A-Lipoic acid Flavonoids L-Carnitine Q-enzyme Q10 Green tea Curcumin Statins Omega-3 fatty acids
Measured in (i) Plasma (ii) Whole blood (iii) White blood cells (iv) Red blood cells (v) Platelets				

TBARS: thiobarbituric acid reactive substances; MDA: malondialdehyde; ox-LDL: oxidized low-density lipoproteins; PCOOH phosphatidylcholine hydroperoxide; ROS: reactive oxygen species; MPO: myeloperoxidase; ADMA: asymmetric dimethylarginine; AOPPs: advanced oxidation protein products; AGEs: advanced glycation end-products; 8-OHdG: 8-hydroxy deoxyguanosine; 8-oxodG: 8-oxo-2′-deoxyguanosine; TAC: total antioxidant capacity; SOD: superoxide dismutase; CAT: catalase; GSH: glutathione; GSH-Px: glutathione peroxidase; GST: glutathione transferase; GSSG: reduced oxidized glutathione; GSH/GSSG: ratio of glutathione/oxidized glutathione; Zn: zinc; Cu: copper; Se: selenium; NAC: N-acetyl-cysteine.
